# A qualitative study to identify barriers to deployment and student training in the use of automated external defibrillators in schools

**DOI:** 10.1186/s12873-017-0114-9

**Published:** 2017-01-19

**Authors:** Line Zinckernagel, Carolina Malta Hansen, Morten Hulvej Rod, Fredrik Folke, Christian Torp-Pedersen, Tine Tjørnhøj-Thomsen

**Affiliations:** 1 Centre for Intervention Research, National Institute of Public Health, University of Southern Denmark, Øster Farimagsgade 5A, 2, DK-1353 Copenhagen, Denmark; 2 The Danish Knowledge Center for Rehabilitation and Palliative care, Department of Oncology, University Hospital Odense and Department of Clinical Research, University of Southern Denmark, Vestergade 17, DK-5800 Nyborg, Denmark; 3 Department of Cardiology, Copenhagen University Hospital Gentofte, Kildegårdsvej 28, DK-2900 Gentofte, Denmark; 4 Duke Clinical Research Institute, 2400 Pratt Street, Durham, NC 27705 USA; 5 Emergency Medical Services, Capital Region of Denmark, University of Copenhagen, Telegrafvej, DK-2750 Ballerup, Denmark; 6 Department of Clinical Epidemiology, Aalborg University Hospital, Hobrovej, DK-9000 Aalborg, Denmark

**Keywords:** Automated external defibrillator, Defibrillators, Cardiopulmonary resuscitation, Resuscitation, Out-of-hospital cardiac arrest, Training, Schools, Technology acceptance model, Implementation

## Abstract

**Background:**

Student training in use of automated external defibrillators and deployment of such defibrillators in schools is recommended to increase survival after out-of-hospital cardiac arrest. Low implementation rates have been observed, and even at schools with a defibrillator, challenges such as delayed access have been reported. The purpose of this study was to identify barriers to the implementation of defibrillator training of students and deployment of defibrillators in schools.

**Methods:**

A qualitative study based on semi-structured individual interviews and focus groups with a total of 25 participants, nine school leaders, and 16 teachers at eight different secondary schools in Denmark (2012–2013). Thematic analysis was used to identify regular patterns of meaning using the technology acceptance model and focusing on the concepts of perceived usefulness and perceived ease of use.

**Results:**

School leaders and teachers are concerned that automated external defibrillators are potentially dangerous, overly technical, and difficult to use, which was related to their limited familiarity with them. They were ambiguous about whether or not students are the right target group or which grade is suitable for defibrillator training. They were also ambiguous about deployment of defibrillators at schools. Those only accounting for the risk of students, considering their schools to be small, and that time for professional help was limited, found the relevance to be low. Due to safety concerns, some recommended that defibrillators at schools should be inaccessible to students. They lacked knowledge about how they work and are operated, and about the defibrillators already placed at their campuses (e.g*.,* how to access them). Prior training and even a little knowledge about defibrillators were crucial to their perception of student training but not for their considerations on the relevance of their placement at schools.

**Conclusions:**

It is crucial for implementation of automated external defibrillators in schools to inform staff about how they work and are operated and that students are an appropriate target group for defibrillator training. Furthermore, it is important to provide schools with a basis for decision making about when to install defibrillators, and to ensure that school staff and students are informed about their placement.

## Background

Out-of-hospital cardiac arrest (OHCA) is a major public health problem that can strike seemingly healthy individuals of any age, often without warning [1]. In Europe, the survival rate following OHCA is only ≈ 10% and costs 350,000 lives each year [2]. It has been known for more than two decades that bystander cardiopulmonary resuscitation (CPR) before the arrival of emergency medical services (EMS) increases survival rates 2–4 times [3, 4]. While CPR can extend the window for successful defibrillation, it is only defibrillation that can re-establish a normal, spontaneous heart rhythm [1], and survival rates >50% have been documented when an automated external defibrillator (AED) is used [5]. However, research suggests that bystanders face barriers in attempting resuscitation, especially concerning the use of AEDs [4, 6].

According to the World Health Organization, the American Heart Association (AHA), and the Institute of Medicine, school-based CPR and AED training are key strategies to increase bystander intervention [1, 2, 7–10]. Training students in CPR and AED use has become mandatory or at least recommended in several countries [2, 7, 10], but even in countries like Denmark that have mandatory resuscitation training in schools, low implementation rates have been observed, particularly for AED training [11, 12]. Previous studies have identified barriers to the implementation of CPR training in school such as perceived need for extensive CPR instruction skills, need for training material, excessive consumption of teaching time, and excessive costs [11–17].

Cardiac arrests in schools are rare events, but the death of a seemingly healthy young person can be especially devastating for the family and local community. Schools with an AED on campus have demonstrated high survival rates for students as well as adults who suffer cardiac arrests [1, 18]. For this reason, there is a growing trend towards placing AEDs in schools [1, 18, 19]. Nevertheless, far from all schools have an AED at campus, and even at campuses with an AED the problems of infrequent application, delayed access to the device, and delayed defibrillation have been observed [1, 20]. Limited funds have been pointed out as the main barrier for widespread implementation [1, 18, 20, 21].

No studies have directed their focus on barriers specifically connected to implementation of AED training of students, and little is known about the barriers to deployment of AEDs at schools. These barriers are likely related to school leaders’ and teachers’ perspectives since they are important actors in the implementation of school changes. Moreover, the national legislation in many countries holds school leaders responsible for ensuring resuscitation training of students and expects teachers to play an important role in the training [11, 15, 22]. Experts do also recommend that teachers conduct the training [7, 23]. The technology acceptance model suggests that the acceptance, adoption, and use of a new technology − in this case the AED − are determined by perceived usefulness and perceived ease of use [24, 25]. By employing the concepts of the technology acceptance model, this study explores school leaders’ and teachers’ perceptions of AEDs to understand more clearly the barriers to implementation of student AED training and AED deployment in schools.

## Methods

### Study design

This qualitative study was based on interviews with school leaders and teachers and was carried out during November 2012 to January 2013 at secondary schools in Denmark (6th- to 9th- grade students, age 12 to 16). We used qualitative methods because our aim was to reach an in-depth understanding of the problem and not to measure factors identified in prior research. Qualitative methods are best suited to elicit meaning and experience from the participants’ point of view and when seeking to describe the complexity of a problem and to reveal issues that the researchers may not have anticipated [26–28].

### Setting

There are approximately 5,6 million people in Denmark and 3500 people are treated annually following OHCA. While the bystander CPR rate has improved significantly from 21% in 2001 to 45% in 2010, the use of defibrillation has remained low at only 2% despite a rapid increase in public access AEDs. In the same period, the 1-year survival increased from 3 to 10% [4]. In 2016, over 14,000 AEDs were registered in the Danish nationwide AED network [29]. There are approximately 2000 secondary schools in Denmark, with around 250,000 students (6^th^-9^th^ grade) and 22,000 teachers [30], and in 2013 about half of schools had an AED deployed at campus [12]. There is no systematic CPR/AED training of school leaders and teachers in Denmark. It is not compulsory at the teachers college [31], and it is up to the individual municipality and school to decide whether to offer such education.

### Sampling of participants

To reach a maximum of variation, we used a strategic sampling strategy to select schools [28], including (1) schools with and without recent experience in CPR training of students, (2) public and private schools, and (3) schools relatively near and far from hospitals. We asked principals to participate in the study, but they could delegate participation to middle managers (e.g., administrative managers and section managers). In the current paper, principals and middle managers are referred to as ‘school leaders’. We sought to recruit one school leader from each school, and between four and eight teachers from some of the participating schools. However, we asked all participating school leaders to select school teachers as we expected that some school leaders would decline to let their teachers participate due to teacher time pressure. Our sample size was determined by data saturation, the point where no, new relevant information appear. The study population comprised a total of 25 participants, nine school leaders and 16 teachers, at eight different schools. The participants varied in age, prior CPR and AED training, and other background variables (Table 1).

**Table 1:** Main characteristics of the study participants

		School leaders *( n* = 9)	Teachers *( n* = 16)
		*n*	*n*
Sex	Women	2	12
	Men	7	4
Age	<55	5	8
	≥55	4	8
	Mean	53 years	46 years
Years teaching	<15	3	9
	≥15	6	7
Years at the school	<10	4	10
	≥10	5	6
CPR course	Yes, incl. AED use	1	3
	Yes, excl. AED use	6	6
	No	2	7
Position	Principal	5	-
	Administrative manager	2	-
	Section manager	2	-
Years as member of the school leadership	<10	4	-
	≥10	5	-
Grade teaching^a^	6th–9th	-	14
	3th–5th	-	4
	10th	-	1

CPR indicates cardiopulmonary resuscitation

AED indicates automatic external defibrillator

^a^The number adds up to more than the participating teachers because they teach different grades

-Indicates that the information is irrelevant for the specific group

### Procedure

The two primary investigators (LZ and CMH) carried out individual interviews with school leaders along with four focus group interviews with three to five teachers in each group from the same school and one individual interview with a teacher. The semi-structured interview protocol was initially developed to explore more broadly barriers to implementation of CPR training in schools [17]. However, we had an interest in the potential of implementing AEDs in schools from the beginning and included a number of predetermined questions on AEDs (Table 2). We were also open and flexible to issues that the interviewees brought up themselves and asked several follow-up questions concerning AEDs. The interview protocol used for school leaders and teachers only differed marginally. The individual interviews lasted 45 minutes to 1½ hour, and each focus group session lasted 1½ to 2 h. All interviews were recorded. Because few of the interviewees had previous AED training, the second author (CMH) displayed an AED after the initial interview in one focus group. We observed and recorded the session and subsequently asked additional questions regarding AEDs. This gave us the opportunity to observe teachers’ interactions with the AED and spontaneous reactions to the AED.

**Table 2:** Semi-structured interview protocol showing the AED specific questions

1) What comes to your mind, when I say AED?
2) What do you know about AEDs?
3) Do you have an AED at the school?
4) Can you imagine AEDs as part of the CPR training of students?
5) What do you think other teachers, students, parents etc. will think about it?
6) Do you have any experiences with AED training of students?
7) What is your opinion about deployment of AEDs in public places?

### Theoretical framework and analysis

The AED is a relatively new technology, and its successful implementation in schools requires school leaders’ and teachers’ acceptance and adoption. The technology acceptance model proposes a theoretical model to explain user acceptance of new technology and identify why the particular technology may be unacceptable [24, 25]. The technology acceptance model was originally developed for the business and information technology sector but has since been applied in medical science [32] and in research on AEDs [33]. According to the technology acceptance model, acceptance of the technology is based on two particular beliefs: perceived usefulness and perceived ease of use. Perceived usefulness is the degree to which a person believes that the new technology will enhance his or her performance of the task at hand. Meanwhile, perceived ease of use is the degree to which a person believes that using the particular technology will be free of effort. By employing the concepts of perceived usefulness and perceived ease of use, we want to explore and describe the manifold conditions that influence school leaders’ and teachers’ acceptance of student training in AED use and AED deployment in schools. The concepts were used in the analytical process as a way to structure the analysis. Thematic analysis was used to identify regular patterns of meaning both within and across the interviews, thus allowing us to specify major themes in the material [28]. The analysis was performed in five steps via an interactive process as shown in Table 3. The qualitative analysis software NVivo10 was used to facilitate the analysis.

**Table 3:** Data analysis procedure

Step 1	The interviews were transcribed (LZ) and read repeatedly by two researchers (LZ and CMH) to gain an overall impression and become familiar with the data’s diversity. The two researchers independently used open coding for each paragraph to discover categories, characteristics, and dimensions of the material [28] and thereafter met to discuss and refine the categories.
Step 2	The categories were discussed with the multidisciplinary research team that included professionals from anthropology (TTT and MHR), medicine (CMH, FF and CTP), and public health (LZ). The coding manual was developed by linking categories into major themes with subthemes (LZ, SMR, TTT, and MH). In this process, we applied the technology acceptance model [24, 25] as a framework for the empirical categories.
Step 3	The data was coded according to the manual (LZ and SMR). As such, the coding was driven by theory as it was organized according to the theoretical categories in the technology acceptance model (e.g., the perceived usefulness of AED training of students and the perceived usefulness of AED deployment at school). But the coding was also data driven as additional themes and subthemes that had emerged from the data were applied (e.g*.,* knowledge and experience with AEDs).
Step 4	Information for each theme was extracted from all of the interviews, and quotes were selected based on how well they illustrated and elucidated the themes (LZ).
Step 5	We looked at relations between the themes

## Results

The results are presented according to three main themes: AEDs as challenging life savers, AED training at schools, and AED deployment at schools. The concepts of ‘perceived usefulness’ and ‘perceived ease of use’ are employed in each of these themes.

### AEDs as challenging life savers

One school leader and three teachers had been trained in how to use an AED. Six school leaders and six teachers had attended a CPR course that did not include AED training, and two school leaders and seven teachers had never taken a CPR course (Table 1). There was a general agreement about public access AEDs as an important initiative with the potential to save lives amongst school leaders and teachers. They recalled stories about people they knew who had died of a cardiac arrest and reflected on whether or not an AED could have saved their lives. Time was identified as a crucial factor. This reality was pointed out most emphatically in areas where the interviewees perceived the arrival time of professional help to be long. AEDs were therefore considered most necessary in those areas. Illustrative of this finding, a teacher stated the following:

*It's insanely important [with AEDs] when you come from here where there isn’t much. If you live in [name of village], it takes several hours before the arrival of an ambulance.* (Teacher 3, School 6, Not AED trained)

Everyone found it valuable to have AEDs located in their neighbourhoods. Also, AEDs were emphasized as being more effective than CPR.

*That is why it’s great with the defibrillator nowadays because it works 10 times better than ventilations and chest compressions, or at least chest compressions, because that is really physically demanding.* (Teacher 4, School 8, AED trained)

Contrary to the shared understanding about the usefulness of AEDs, there was a general uncertainty and insecurity regarding the ease of use of AEDs among school leaders and teachers. This seemed to be closely connected with their level of knowledge about AEDs and familiarity with them, which was generally low. The majority of the interviewees were unaware of how AEDs work and how they are operated. For instance, that it only delivers a shock when needed, or that it provides users with detailed, step-by-step, real-time spoken instructions. As a school leader described it when he was asked how he thought it would be to use an AED,

*I have no idea. I should say. The courses I have taken haven’t [included AED training]. I don’t know. I have no idea. I wouldn’t know how to use it. I would hope for a written instruction to be able to do a little.* (School Leader, School 7, Not AED trained)

Another contributing factor to this was school leaders’ and teachers’ knowledge about AEDs traditionally being used by health professionals only. Most would feel uncomfortable using an AED without prior instruction, and some even expected training to be necessary for using one.

The nature of a cardiac arrest situation was closely connected to the reason why school leaders and teachers found AED training to be important. They described a cardiac arrest situation as being extreme, since you are dealing with life and death in such circumstances, and every minute counts. They therefore expected that most people, including themselves, would panic in such a situation. The school leaders and teachers also stated that they would try to apply the AED on a patient, but worried about whether or not they would act inappropriately without prior AED training. Many (of those without prior AED training) expected that they would need to read instructions before using AEDs and expressed concerns about reading time and if they would manage to read and remember the instructions in such a situation. This finding is illustrated by a teacher saying,

*I think you should be familiar with it [the AED] because if you have to study it on the wall in a panic situation, I think it would not be particularly smart. It wouldn’t be smart for me. I would run around in circles. I wouldn’t even remember one line.* (Teacher 2, School 7, Not AED trained)

The AED was considered a powerful device. This contributed to the school leaders’ and teachers’ perception of the AED as being useful yet having a negative image regarding perceived ease of use. Accordingly, several leaders and teachers had safety concerns about the AED. They believed that AEDs could be potentially dangerous for the person in cardiac arrest and for the bystanders, and they feared that AEDs could cause more harm than good if used inappropriately. As one teacher said,

*It [the AED] may not do any good if something has to be done in a special way. Otherwise, you risk causing harm. That was not the intention. It was the opposite you wanted to achieve.* […] *It is not of any use that you have the best intentions but end up killing the man.* (Teacher 2, School 7, Not AED trained)

The few who had received AED training emphasized several positive qualities about AEDs related to their ease of use. For instance, they said that it guides one by telling one what to do, it does not forget what to do, it decides what should be done (e.g*.,* shock/not-shock), it cannot cause harm, etc. They went on to explain how these qualities could generate support, comfort, and reassurance, and take away some responsibility from the bystander. Receiving AED training appeared to be a turning point for the interviewees as the training resulted in new realization of the ease of use of AED. Thus, the AED was subsequently viewed as easier to use and not being potentially harmful. This idea is illustrated by a school leader who explained how the AED training changed her perception of it:

*Before I attended all this [CPR/AED training], I'm not sure I would dare to open it [the AED]. Because will it provide a shock immediately, or what does it do, or should I be careful not to touch it with two hands, or should I sit down, or should I… There are so many things with this device where you would think, Hmmm.* (School Leader, School 8, AED trained)

When we instructed a group of teachers in the use of the AED, they were surprised at how small and handy it was and that it was simple to use and not dangerous at all. Hence, even a little information appeared to be of significance regarding the AED’s perceived ease of use. Indeed, many school leaders and teachers changed their perception of AEDs when the interviewers explained how it works. For example, the fact that the AED cannot cause harm was commented on by one leader:

*Just that information is enough to … Now you are not nervous of doing anything wrong. But when you don’t know better, then you think*, Wow! (Teacher 2, School 7, Not AED trained)

However, knowledge and training did not seem to change school leaders’ and teachers’ perception of the AED supposedly belonging to a category different than CPR due to it being a technical device. When referring to the AED, they used words such as ‘machine’, ‘technical’, ‘device’, and ‘funny computer voice’. The technical aspect of the AED created distance towards it, and for some, insecurity about using it. Illustrative of this finding, a school leader stated,

*It is limited how much wrong you can do when providing rescue breathings or artificial ventilation and things like that. And it’s probably, or it is the same with the AED. But now you are suddenly applying a device instead of yourself and doing something to another human being.* (School Leader, School 5, Not AED trained)

Thus, knowledge and AED training seemed to be crucial for school leaders’ and teachers’ perception of the ease of use of and potential harm by AEDs, yet the insecurity caused by the technical aspect of the AED remained.

### AED training at schools

Two schools currently trained their students how to use AEDs as part of a CPR course, an additional two had implemented CPR training without AED training, three schools had not implemented AED or CPR training, and at one school, the school leader did not know whether or not there was any kind of CPR training (Table 4). The training seemed to be systematic in only one school by ensuring that all students had received CPR and AED training before graduating from secondary school. Meanwhile, school leaders and teachers perceived AED training in schools as being useful for three main reasons. First, it was considered natural and desirable to follow the technological development. They were aware the AED had become part of guidelines for basic life support, and they therefore found it valuable and natural to incorporate it into CPR training of students. As a teacher said:

**Table 4:** School characteristics

School	School type	No. of interviewees	CPR training of students	AED training of students	AED installed	Location of the AED	Number of students^a^	Distance from a hospital^b^	Urban or rural
1	Public	1 school leader	Yes	Yes	Yes	School and sport facility	1050	2 km	Urban
2	Private	1 school leader and 1 teacher	No	No	No	-	400	1 km	Urban
3	Public	1 school leader and 5 teachers	No	No	Yes	Sport facility	700	16 km	Rural
4	Public	1 school leader	School leader does not know	School leader does not know	Yes	Sport facility	515	23 km	Rural
5	Private	1 school leader	Yes	No	No	-	210	1 km	Urban
6	Public	2 school leader and 3 teachers	Yes	No	Yes	School	860	20 km	Rural
7	Public	1 school leader and 3 teachers	No	No	Yes	Sport facility	350	20 km	Rural
8	Public	1 school leader and 4 teachers	Yes	Yes	Yes	Sport facility	650	35 km	Rural

CPR indicates cardiopulmonary resuscitation

AED indicates automatic external defibrillator

- Indicates that the information is irrelevant for the specific school

^a^Including all grades at the school, 0^th^–10^th^ grade

^b^Perceived distance to professional help

*It is only natural that it [the AED] is part of the [resuscitation] training. You have to follow the development. And if that’s what it takes to save more lives, then it's just fine.* (Teacher 1, School 1 AED trained)

Second, the interviewees emphasized that the increasing deployment and visibility of AEDs have made it relevant to learn about AEDs because the chance of getting to use one had increased. This idea was illustrated by a teacher who explained why students should be trained in using an AED:

*It’s natural, because there are more and more AEDs around. Thus, it would be completely illogical to do without it.* (Teacher 3, School 8, AED trained)

Third, school leaders and teachers expected that AED training would have a positive effect. Those who had experience with AED training of students explained how the training made students familiar and confident with it so that they would actually dare to use one. This was commented on by a school leader, who said,

*It might have been red, the one they [the students] trained with, but this one is green, but they will still remember to open it up and that it says something where others might not dare to open it because, ‘Does it shock me? What happens?’ Just the fact that they have heard about it, have received the training here in school, is enough for them to dare to start up.* (School Leader, School 8, AED trained)

Nonetheless, some school leaders and teachers were ambiguous about students being the target group for AED training and currently did not feel confident about implementing AED training. The ambiguity is evident in the following answer from a school leader when asked if he could imagine AEDs as part of the CPR training of students:

*Regarding training of the children, I actually do not know. I would say, ‘Let me see how relevant is it for the students and which age group’ … There is no need to train children in kindergarten who don’t understand what this is all about. You know about it. Would 8* ^*th*^ *and 9* ^*th*^ *grade be appropriate? Would they be able to use it?* (School Leader, School 7, Not AED trained)

School leaders’ and teachers’ ambiguity about implementing AED training in schools was closely related to their perceived ease of use of AEDs (or lack thereof) with a perception of the AED as being potentially dangerous, technical, and thus difficult to use. At the same time, they recognized the usefulness of the AED both in general and in the training of students and acknowledged their limited knowledge about AEDs. This mindset led some interviewees to request expert opinions on whether or not it would be appropriate to train secondary school students in the use of AEDs, and if so, they sought directions on what grade would be suitable. Others recommended that AED training be implemented at grades higher than CPR training, and some suggested that it should take place in high schools as they found secondary school students to be too young. School leaders’ and teachers’ safety concerns about AEDs troubled them especially regarding student safety during AED training and whether or not the students were too young to be trusted with an AED. This is illustrated by a school leader who said,

*Hmm … There are a few [students], I would rather not see with an AED in their hands. I don’t think John should try that. No, I cannot imagine that. I think it needs to be in high school.* (School Leader, School 5, Not AED trained)

Furthermore, some interviewees projected their own insecurities with the technical aspect of AEDs on to the students, expecting them to be apprehensive towards using them also AED trained interviewees:

*I think it has something to do with… That when it is rescue breathings and chest compressions, it is such a hands-on thing where the AED is a machine, which they [the students] have to pull down from the wall, and then it has such a funny computer voice. I think they will distance themselves more from it because it is a machine. But it’s only a hunch. I don’t know at all.* (Teacher 3, School 3, AED trained)

Contrary to this perception, some interviewees argued that students possess better technical skills than adults because they are more exposed to technology in their everyday lives. The AED was therefore believed to be easier for them to use as illustrated by a teacher saying the following:

*I really believe that the technical use of it [the AED]… I think children would be able to do that and many of them even faster than us. That is what we can see with everything else, they get in their hands. Pacifiers in their mouths and up we go in front of the computer.* (Teacher 2, School 7, Not AED trained)

The few school leaders and teachers with practical experience in AED training of students did not distinguish between CPR and AED training and had not experienced special reactions from students regarding AEDs. Two teachers explained this situation when asked about students’ reactions to AED training:

*It's not like there is a special reaction [from the students] when it is about AEDs [compared to CPR training].* (Teacher 5, AED trained)

The interviewees (both AED trained and not AED trained) emphasized the need for the AED to be included in CPR-training material in order for it to become a natural part of resuscitation training. Those using such materials also explained how this had facilitated the AED to be included in the training.

### AED deployment at schools

Six schools had an AED installed at their campuses. Two were located specifically at the school, and the others were located at their sports’ facilities (Table 4). None of the deployed AEDs had been used according to the interviewees. In the following section, challenges with the installed AEDs and barriers to deployment of AEDs at schools will be presented. School leaders and teachers had limited knowledge about the AEDs already installed at their campuses since they had not been informed about the AED placement. Consequently, not all interviewees knew there was an AED at their schools. Accessibility to the AEDs was another issue. For instance, school leaders and teachers discussed whether or not one should break a sealing or if they needed a key to open the box with the AED inside. They also wanted to know if the AEDs located at the sports facilities were inaccessible to them because only sports’ teachers had a key, and they were concerned about the distance from the AED to different parts of the schools. The concerns about accessibility to the AEDs are illustrated in the following fragment originating from one of the focus groups:

*I have thought about… I see it [the AED] every morning. I wonder if it is locked. It surely cannot be locked if I suddenly need it immediately.* (Teacher 2, Not AED trained)

*I just think that there is a long distance to the oldest students.* (Teacher 3, Not AED trained)

*It’s something like, I start with chest compressions, and then another one has to run to get it.* (Teacher 2)

*You just have to find someone who has the key if it’s locked.* (Teacher 1, Not AED trained)

*It better not be. You can see how little we know about it. We know nothing about it.* (Teacher 2)

*I suppose it’s sealed like our fire equipment.* (Teacher 1)

(School 6)

However, the interviewees wish for the AEDs to be accessible did not apply to students. School leaders and teachers expected and recommended the AEDs to be placed where students could not reach them such as in the secretary’s office. This was due to safety concerns regarding the AED and concerns about if children can be trusted with one of these devices. As one of the teachers said,

*I'm not sure I would dare to have it [the AED] placed so they [the students] could access it.* (Teacher 3, School 7, Not AED trained)

There were discrepancies concerning how useful the deployment of AEDs is at schools. Although none of the interviewees were against such an initiative, they disagreed on the relevance. While some found it crucial to place AEDs at schools, others thought it was overblown. Their considerations were centred on the probability of the AED being used hereunder (1) the risk of a cardiac arrest, (2) the number of people at the school, and (3) the time of arrival of professional help. School leaders and teachers considered students to be at low risk of suffering a cardiac arrest. Most interviewees also had considerations about the risk of their colleagues, the students’ parents, those using the school after class time (often senior citizens), and their own risk. All of these issues were considered to be of a higher risk than the students.

*If we should have one [an AED], I always think like that in a school such as ours. I don’t know. Someone may be able to say whether that is reasonable. The probability of it being used with 400 children is not very big. However, there are many adults. I would probably be the first.* (School Leader, School 2, Not AED trained)

The interviewees stated that the more people at the school, the more likely a cardiac arrest would occur and that an AED would be needed. They therefore reflected on the size of the school and on how many people gathered at the school during the day. This idea is illustrated by a teacher declaring,

*It has also something to do with the size of the school. I cannot understand why there isn’t an AED in a school or at other institutions where there are so many people.* (Teacher 2, School 7, Not AED trained)

Interviewees at schools located relatively far from hospitals found it highly relevant to deploy AEDs at their schools. Conversely, at schools located near a hospital, interviewees perceived time to professional help to be short, so having an AED at the school was not expected to result in any additional value. One leader stated accordingly,

*Perhaps it would be a little bit of overkill [to have an AED]. We have a hospital over there and another hospital is 45 s away if they are fast. That should also be taken into account.* (School Leader, School 5, Not AED-trained)

School leaders and teachers found the usefulness of AED deployment at schools to be low if they did not take others’ risk of having a cardiac arrest into account (other than students), considered their school to be relatively small, and perceived time to professional help to be short (based on distance from the nearest hospital). They did not reject the idea but requested expert opinions on whether or not they should have AEDs placed at their school. Their considerations on this matter did not seem to be influenced by prior AED training. Nonetheless, one teacher expressed that it would be useless to have an AED at the school if no one were trained in how to use one. The interviewees only rarely mentioned the cost of AEDs as a barrier for placing them in schools. In one focus group, teachers had not received an AED after requesting their school leader for one, and they believed the costs had been decisive but had not been given an explanation. A school leader at another public school asked the interviewer about the price of an AED, and when the interviewer gave him an approximate price, he stated that this was no obstacle for the school to purchase a couple of AEDs.

## Discussion

### Main findings

School leaders and teachers are concerned with the perceived ease of use of AEDs. They believe that AEDs are potentially dangerous, overly technical, and difficult to use. This was related to their familiarity with AEDs, which was generally low. For instance, few knew how AEDs work and are operated. They were therefore ambiguous about whether or not secondary school students (6^th^- to 9^th^-grade students, ages 12 to 16) are the right target group or which grade is suitable for AED training, even though they perceived the training to be useful. School leaders and teachers were also ambiguous about AED deployment at schools, which was rooted in their perceived usefulness of such an initiative. Those only accounting for the risk of cardiac arrest among students, who considered their schools to be small and thought that the time of arrival of professional help was short, found the relevance of AEDs at schools to be low. Furthermore, due to the perception of the AED being potentially dangerous, some recommended that AEDs at schools should be unreachable to students. Interviewees lacked information on the AEDs placed on their campus (e.g., how to access the devices). Prior training and even a little knowledge about AEDs (e.g., that they cannot cause harm) were crucial for school leaders’ and teachers’ perceptions of AED training of students but not for their considerations on the relevance of AED placement at schools.

### Discussion of findings

#### AED training of students

We found that school leaders and teachers are concerned about the ease of use of AEDs including the safety aspect of AEDs and the issue of AEDs being technical and difficult to use. This mindset resulted in uncertainty about what student grades should receive AED training and if secondary school students were too young to be trained in how to use an AED. Several studies have demonstrated that AEDs can be safely and effectively used by people with little or no prior training [34–37] and also by children [23]. In simulation training studies, even children of 6–7 years have been found capable of quickly delivering a shock with minimal training [38]. However, there are no clear recommendations on what grade should undergo AED training in schools, and no studies have extensively discussed at what age children should be encouraged to use AEDs in real-life situations [23]. It is also noteworthy that medical experts have previously had the same concerns towards AEDs and have urged caution against allowing AEDs to be used by the general public [39]. It was not until 2000 that the AHA clearly acknowledged the value of AEDs and their safety for lay rescuers [40]. Thus, it is not surprising that school leaders and teachers have reservations towards AED training of students, especially given their limited knowledge of AEDs and the lack of clear guidelines on an appropriate target group for AED training. According to our study, AED training and information about how AEDs work and are operated is crucial since even minimal knowledge was critical for their perception of AED student training. On the other hand, the technical aspect of AEDs caused insecurity among some school leaders and teachers, and they therefore expected students to be apprehensive towards AED training. This insecurity did not seem to be affected by previous AED training. School leaders and teachers with actual experience with AED training of students had not experienced any special reactions from students regarding the AED.

#### AED deployment

We found that school leaders and teachers disagreed on the usefulness of AEDs placed at schools. None rejected the idea but requested expert opinions on whether or not it was relevant and questioned if they should have an AED at their school. The AHA recommends the deployment of AEDs in schools with a documented need, that is, schools with at least one of the following criteria: (1) frequency of use is such that there is reasonable probability of use within 5 years, (2) children or adults attend the school who are thought to be at high risk for cardiac arrest, and (3) an EMS call-to-shock interval of <5 min cannot be reliably achieved [41]. The contents of these recommendations resemble school leaders’ and teachers’ considerations about the relevance of AED placement at schools. We found that these were centred on the probability of the AED being used (risk assessment of cardiac arrest, number of people at the school, and evaluation of time to professional help). However, it was unclear as to how interviewees evaluated these factors, for instance, what they based their risk assessment on and when they considered time of arrival of professional help to be too long. Moreover, their estimation of EMS response time was misunderstood, for they based this on distance to the nearest hospital and not to the nearest EMS station. We found that perceived usefulness of the AED in the minds of school leaders and teachers was the main challenge for deployment of AEDs at schools. In the literature, there seems to be consensus regarding lack of funding as the primary obstacle, which was built upon several quantitative studies that cited this issue to be the most frequent barrier [1, 18]. However, our study could not confirm these findings since the cost of AEDs were rarely mentioned as a barrier for placing AEDs at schools. The perceived relevance of AEDs at schools (or lack thereof) may have been an underlying mechanism for claiming lack of funding as a barrier in previous studies. After all, this idea might have influenced interviewees’ thoughts on how to prioritize finances and time to apply for funding. Our study further demonstrates challenges with AEDs already installed at schools since school leaders and teachers lack information about location, accessibility, and proper use of AEDs. Many do not know if they can access AEDs or know how to access them and are uncertain about how to use them. They are also insecure about using them without prior instruction, and some are even unaware of the presence of the AEDs in their schools. In line with this, previous studies among American college and university students have demonstrated that the vast majority lacked knowledge of the location of AEDs within their own campuses [42, 43]. We also found that school leaders and teachers recommended that AEDs at schools be unreachable to students due to safety concerns. This may, to some extent, explain why previous studies have found that even with an AED installed at the school, AED application was infrequent [20, 44].

### Implications

In order to strengthen implementation of student AED training, it is important to disseminate information on how AEDs work and are operated. In addition, AED training should be incorporated in CPR courses of school staff, and staff members should be informed that school students are appropriate recipients of AED training. Future studies should investigate at what age AED training in schools is most suitable and at what age children should be encouraged to use AEDs in real-life situations. To reach successful implementation of AED deployment in schools, schools should be provided with a basis for decision-making about when to install an AED. Authorities should thus provide clear recommendations such as those by the AHA [41]. It is additionally important to prepare schools for how to assess such criteria (e.g., if there are children or adults at the school who are at high risk of cardiac arrest). Schools should also be informed about the importance of ensuring easy access and information to the entire school staff and students about AEDs already installed at their facilities. Our findings provide a strong basis for future quantitative studies which may investigate the generalizability of this study. Together with this study, it would provide solid knowledge to be disseminated to schools and decision makers and to be used as a basis to develop new interventions and initiatives seeking to improve implementation of student AED training and AED deployment in schools. Importantly, our findings also contribute with insight into more general aspects of AED implementation such as the consistently low use of public access AEDs [4, 6] as many of the barriers identified in our study reach beyond the school setting. We found that it is of utmost importance to familiarize lay persons to AEDs. This is supported by a recent study suggesting that prior knowledge and hands-on AED training promoted confidence and prevented panic among lay bystanders in real cardiac arrest situations [45]. Thus, in addition to ensuring placement, accessibility, and visibility of AEDs, it is important to increase bystander willingness to use them. Successfully implementing student training in AED use will contribute to reaching this goal by ensuring that the general public has been familiarized with AEDs.

### Strengths and limitations

By using qualitative methods, we were able to reach an in-depth understanding of why implementation of AED training of students has been unsuccessful in Denmark, and to describe school leaders’ and teachers’ complex and ambiguous relationships towards the deployment of AEDs at schools. The character of qualitative research has also been pointed out to contribute to a broader and improved understanding within medical research, including implementation science [26, 27]. We managed to obtain a broad representation of schools and interviewees, successfully portrayed different positions, and reached data saturation, which is defined as the point at which no new relevant data emerges [28]. Nevertheless, we acknowledge that it can be difficult to determine data saturation, and that it is possible that our understanding could have been expanded if we had conducted more interviews with a wider variety of teachers and shool leaders. Also, the perceptions of AEDs in general could have changed since the interviews were conducted in 2012–2013. Furthermore, context-specific considerations should, as always [28], be taken into account when applying our findings to other settings. This is because the Danish school system or the public’s knowledge and ideas regarding AEDs cannot be generalized for all communities.

## Conclusions

School leaders and teachers are ambiguous about whether or not secondary school students is an appropriate target group and do not know what grade is suitable for AED training. This was connected to their perceived ease of use of AEDs. They were also ambiguous about AED deployment at schools, which was rooted in their perceived usefulness of such an initiative, and some recommended that AEDs at schools should be unreachable to students. Moreover, school leaders and teachers only had limited knowledge about the AEDs installed at their schools. Prior training and even a little knowledge about AEDs (e.g., that they cannot cause harm) were crucial for their perceptions of AED training of students but not for their considerations on the relevance of AED placement at schools. In light of our findings, the successful implementation of AEDs in schools (both student AED training and AED deployment) requires school staff to be informed about how AEDs works and that AEDs cannot cause harm. They also must be trained in AED use. Authorities should systematically inform schools that students make for an appropriate target group for AED training and should also provide schools with a basis for decision-making about when to install AEDs. Finally, school staff and students should be informed of the location and accessibility of AEDs deployed in their schools.

## Abbreviations

AED: Automated external defibrillator
AHA: American Heart Association
CPR: Cardiopulmonary resuscitation
EMS: Emergency medical services
OHCA: Out-of-hospital cardiac arrest

## References

[1] Institute of Medicine (IOM) (2015). Strategies to Improve Cardiac Arrest Survival: A Time to Act.

[2] Georgiou M, Lockey AS (2013). ERC initiatives to reduce the burden of cardiac arrest: the European Cardiac Arrest Awareness Day. Best Pract Res Clin Anaesthesiol.

[3] Herlitz J, Svensson L, Holmberg S, Angquist KA, Young M (2005). Efficacy of bystander CPR: intervention by lay people and by health care professionals. Resuscitation.

[4] Wissenberg M, Lippert FK, Folke F, Weeke P, Hansen CM, Christensen EF, Jans H, Hansen PA, Lang-Jensen T, Olesen JB (2013). Association of national initiatives to improve cardiac arrest management with rates of bystander intervention and patient survival after out-of-hospital cardiac arrest. JAMA.

[5] Valenzuela TD, Roe DJ, Nichol G, Clark LL, Spaite DW, Hardman RG (2000). Outcomes of rapid defibrillation by security officers after cardiac arrest in casinos. N Engl J Med.

[6] Hansen CM, Lippert FK, Wissenberg M, Weeke P, Zinckernagel L, Ruwald MH, Karlsson L, Gislason GH, Nielsen SL, Kober L (2014). Temporal trends in coverage of historical cardiac arrests using a volunteer-based network of automated external defibrillators accessible to laypersons and emergency dispatch centers. Circulation.

[7] Bohn A, Lukas RP, Breckwoldt J, Bottiger BW, Van Aken H (2015). ‘Kids save lives’: why schoolchildren should train in cardiopulmonary resuscitation. Curr Opin Crit Care.

[8] Bottiger BW, Van Aken H (2015). Training children in cardiopulmonary resuscitation worldwide. Lancet.

[9] Cave DM, Aufderheide TP, Beeson J, Ellison A, Gregory A, Hazinski MF, Hiratzka LF, Lurie KG, Morrison LJ, Mosesso VN (2011). Importance and implementation of training in cardiopulmonary resuscitation and automated external defibrillation in schools: a science advisory from the American Heart Association. Circulation.

[10] Lockey AS, Georgiou M (2013). Children can save lives. Resuscitation.

[11] Hart D, Flores-Medrano O, Brooks S, Buick JE, Morrison LJ (2013). Cardiopulmonary resuscitation and automatic external defibrillator training in schools: “is anyone learning how to save a life?”. CJEM.

[12] Hansen CM, Zinckernagel L, Ersbøll AK, Tjørnhøj-Thomsen T, Wissenberg M, Lippert FK, Weeke P, Gislason GH, Køber L, Torp-Pedersen C et al. Rates of, Barriers and Facilitators to Cardiopulmonary Resuscitation Training in Schools Following Eight Years of Mandating Legislation: a nationwide survey. 2017.

[13] Lafferty C, Larsen PD, Galletly D (2003). Resuscitation teaching in New Zealand schools. N Z Med J.

[14] Miro O, Jimenez-Fabrega X, Espigol G, Culla A, Escalada-Roig X, Diaz N, Salvador J, Abad J, Sanchez M (2006). Teaching basic life support to 12–16 year olds in Barcelona schools: views of head teachers. Resuscitation.

[15] Mpotos N, Vekeman E, Monsieurs K, Derese A, Valcke M (2013). Knowledge and willingness to teach cardiopulmonary resuscitation: a survey amongst 4273 teachers. Resuscitation.

[16] Reder S, Quan L (2003). Cardiopulmonary resuscitation training in Washington state public high schools. Resuscitation.

[17] Zinckernagel L, Malta Hansen C, Rod MH, Folke F, Torp-Pedersen C, Tjornhoj-Thomsen T (2016). What are the barriers to implementation of cardiopulmonary resuscitation training in secondary schools? A qualitative study. BMJ Open.

[18] Drezner JA, Toresdahl BG, Rao AL, Huszti E, Harmon KG (2013). Outcomes from sudden cardiac arrest in US high schools: a 2-year prospective study from the National Registry for AED Use in Sports. Br J Sports Med.

[19] Watson AM, Kannankeril PJ, Meredith M (2013). Emergency response planning and sudden cardiac arrests in high schools after automated external defibrillator legislation. J Pediatr.

[20] Rothmier JD, Drezner JA, Harmon KG (2007). Automated external defibrillators in Washington State high schools. Br J Sports Med.

[21] Kovach J, Berger S (2012). Automated external defibrillators and secondary prevention of sudden cardiac death among children and adolescents. Pediatr Cardiol.

[22] Undervisningsministeriet (2009). Fælles Mål 2009 Færdselslære. Faghæfte 20. Undervisningsministeriets håndbogsserie.

[23] Plant N, Taylor K (2013). How best to teach CPR to schoolchildren: a systematic review. Resuscitation.

[24] Davis FD (1989). Perceived Usefulness, Perceived Ease of Use, and User Acceptance of InformationTechnology. MIS Q.

[25] Davis A, Bagozzi RP, Warshaw PR (1989). User Acceptance of Computer Technology: A Comparison of Two Theoretical Models. Manag Sci.

[26] Curry LA, Nembhard IM, Bradley EH (2009). Qualitative and mixed methods provide unique contributions to outcomes research. Circulation.

[27] Malterud K (2001). The art and science of clinical knowledge: evidence beyond measures and numbers. Lancet.

[28] Patton M (2002). Qualitative Research & Evaluation Methods.

[29] The Danish Foundation TrygFonden. The Danish AED Network. https://hjertestarter.dk/find-hjertestartere/find-hjertestartere. Accessed 15 Sept 2016.

[30] Ministry for Children, Education and gender equality. Statistics on public and private schools (in Danish). https://www.uvm.dk/Service/Statistik/Statistik-om-folkeskolen-og-frie-skoler. Accessed 15 Sept 2016.

[31] Ministry for children, education and gender equality. Ministerial order on the Bachelor of Science Schoolteachers 2015 (In Danish). https://www.retsinformation.dk/forms/R0710.aspx?id=174218. Accessed 15 Sept 2016.

[32] Strudwick G (2015). Predicting nurses’ use of healthcare technology using the technology acceptance model: an integrative review. Comput Inform Nurs.

[33] Money AG, Barnett J, Kuljis J (2011). Public claims about automatic external defibrillators: an online consumer opinions study. BMC Public Health.

[34] Moore JE, Eisenberg MS, Cummins RO, Hallstrom A, Litwin P, Carter W (1987). Lay person use of automatic external defibrillation. Ann Emerg Med.

[35] Mosesso VN, Shapiro AH, Stein K, Burkett K, Wang H (2009). Effects of AED device features on performance by untrained laypersons. Resuscitation.

[36] Yeung J, Okamoto D, Soar J, Perkins GD (2011). AED training and its impact on skill acquisition, retention and performance--a systematic review of alternative training methods. Resuscitation.

[37] Bhanji F, Donoghue AJ, Wolff MS, Flores GE, Halamek LP, Berman JM, Sinz EH, Cheng A (2015). Part 14: Education: 2015 American Heart Association Guidelines Update for Cardiopulmonary Resuscitation and Emergency Cardiovascular Care. Circulation.

[38] Uray T, Lunzer A, Ochsenhofer A, Thanikkel L, Zingerle R, Lillie P, Brandl E, Sterz F (2003). Feasibility of life-supporting first-aid (LSFA) training as a mandatory subject in primary schools. Resuscitation.

[39] Harrison-Paul RS (2009). Training Laypeople to use Automatic External Defibrillators: Are all of their needs being met? PhD thesis.

[40] The American Heart Association in Collaboration with the International Liaison Committee on Resuscitation. Guidelines 2000 for Cardiopulmonary Resuscitation and Emergency Cardiovascular Care. Part 4: the automated external defibrillator: key link in the chain of survival. Circulation 2000;102(8 Suppl):I60–76. 10966663

[41] Hazinski MF, Markenson D, Neish S, Gerardi M, Hootman J, Nichol G, Taras H, Hickey R, O’Connor R, Potts J (2004). Response to cardiac arrest and selected life-threatening medical emergencies: the medical emergency response plan for schools: A statement for healthcare providers, policymakers, school administrators, and community leaders. Circulation.

[42] Rega P, Fink B (2015). AED Education: A Dilemma for Public Health and a Challenge for Critical Care Specialists. J Intensive Crit Care.

[43] Bogle B, Mehrotra S, Chiampas G, Aldeen AZ (2013). Assessment of knowledge and attitudes regarding automated external defibrillators and cardiopulmonary resuscitation among American University students. Emerg Med J.

[44] Swor R, Grace H, McGovern H, Weiner M, Walton E (2013). Cardiac arrests in schools: assessing use of automated external defibrillators (AED) on school campuses. Resuscitation.

[45] Hansen CM, RosenKranz SM, Folke F, Zinckernagel L, Tjørnhøj-Thomsen T, Torp-Pedersen C, Sondergaard KB, Nichol G, Rod MH: Lay Bystanders’ Perspectives on What Facilitates Cardiopulmonary Resuscitation and Use of Automated External Defibrillators in Real Cardiac Arrests. 2017. 10.1161/JAHA.116.004572 PMC5524003 28288975

[46] The Danish Data Protection Agency. Danish Act on Processing of Personal Data. https://www.datatilsynet.dk/english/the-danish-data-protection-agency/introduction-to-the-danish-data-protection-agency/. Accessed 22 Nov 2016.

